# The decision delivery interval in emergency caesarean section and its associated maternal and fetal outcomes at a referral hospital in northern Tanzania: a cross-sectional study

**DOI:** 10.1186/s12884-017-1608-x

**Published:** 2017-12-07

**Authors:** Birjna A. Hirani, Bariki L. Mchome, Nicholaus S. Mazuguni, Michael J. Mahande

**Affiliations:** 1 Department of Obstetrics and Gynecology, Kilimanjaro Christian Medical Centre, Moshi, Tanzania; 2 Department of Epidemiology & Biostatistics, Institute of Public Health, Kilimanjaro Christian Medical University College, Moshi, Tanzania; 3 Kilimanjaro Christian Medical University College, Moshi, Tanzania

**Keywords:** Emergency caesarean section, Decision delivery interval (DDI), Pregnancy outcomes

## Abstract

**Background:**

Decision delivery interval (DDI) is the time line between a decision to conduct an emergency caesarean section and actual delivery of the baby. Prolong DDI constitute a third phase delay in provision of emergency obstetric care. Intervention designed to minimize DDI are vital, in attempt to prevent maternal morbidity and neonatal morbidity and mortality. The feasibility and practicability of the recommended DDI in recent studies have been questioned especially in limited resource setting and therefore the objective of our study was to determine the DDI and its associated fetalmaternal outcomes at a tertiary referral hospital in Tanzania.

**Methods:**

This was a retrospectivecross-sectional study of inpatient cases who underwent emergency caesarean section from January to September 2014. Data were collected from birth registry and case files of patients. Data analysis was performed using statistical package for social science (SPSS) version 22.0. Odds ratio (ORs) and 95% confidence interval for maternal and fetal outcomes associated with DDI were estimated using Logistic regression models. A *p*-value of less than 5% was considered statistically significant.

**Results:**

A total of 598 women who underwent emergency caesarean section were recruited. The median Decision Delivery Interval was 60 min [IQR 40–120]. Only 12% were operated within 30 min from decision time. Shortest DDI was seen in patients with Cephalopelvic Disproportion (CPD) and uterine rupture (40 min and 45.5 min) as compared to other conditions. Cases with impending uterine rupture, cord prolapse, APH and fetal distress showed to have shorter DDI. There was no significant association between DDI and neonatal transfer,1st and 5^th^minute Apgar score, maternal blood loss (OR: 5.79; 95% CI 0.63–1.64) and hospital stay (OR: 1.02; 95% 0.63–1.64).

**Conclusions:**

Contrary to the recommended DDI by ACOG & AAP of 30 min is not feasible in our setting, time frame of 75 min could be acceptable but clinical judgment is required to assess on the urgency of caesarean section in order to prevent maternal and neonatal morbidity and mortality.

## Background

Emergency caesarean section is a type of surgical procedure which is performed when there is an immediate threat to the life of fetus or woman during delivery [1]. According to the American College of Obstetrics and Gynecology (ACOG)& American Academy of Pediatrics (AAP) recommendation, the emergency caesarean section should be performed in a time phase of 30 min from the decision to conduct it [2]. Therefore Decision to Delivery interval (DDI) is the time interval from decision made to perform an emergency caesarean section till the delivery of the baby [1].

Due to overburdened and weak health systems that characterize low income countries often mean the DDI is stretched to 75 min without any significant morbidities [3]. However if the DDI is pushed over 75 min a significant maternal and neonatal morbidities and mortalities are likely to occur [4]. A recent study in Nigeria by Bello and colleagues [4] reported 5.1% perinatal mortality that was statistically significant with increase in DDI beyond 75 min. Other composite outcomes that have been attributed to increased DDI by previous authors include 1% of both fresh and macerated still births,5%.

Apgar score less than 7 in the fifth minute and 3% died in neonatal ICU [5].

Previous investigators also have shown that the standard DDI is not met in many emergency obstetric units and longer duration of DDI of more than 75 min have shown an impact on maternal and neonatal outcomes [3]. In addition, various controversies have risen for the optimal duration of DDI [6]. Since this important duration is being proposed by international professional organization as a benchmark for the standard duration to conduct emergency caesarean section, it therefore becomes important to validate its usage in different clinical setup before it’s universally accepted.

Therefore, this study aimed to determine the decision delivery interval and its associated fetal and maternal outcome at Kilimanjaro Christian Medical Centre tertiary hospital in Tanzania.

## Methods

### Study design and setting

This was a retrospective cross sectional study conducted at Kilimanjaro Christian Medical Centre (KCMC), Moshi, Tanzania. KCMC is one of the four consultant referral hospitals in Tanzania which is located in Kilimanjaro region in the Northern zone of Tanzania. The hospital has an obstetric unit with a labor ward in the same floor with one operating theatre for elective and emergency cesarean section. The unit has around 4000 deliveries annually including inpatient and referrals.

### Study subjects

Study subjects consisted of inpatient women who underwent emergency caesarean section from 1st January to 30th September 2014. These were pregnant women who were admitted in the antenatal ward for obstetric care and later underwent emergency caesarean section. Women with incomplete records on delivery model and those referred from other health facility for emergency obstetric care were excluded. Those referred from other health facilities for emergency obstetric care including emergency caesarean section were excluded to reduce first and second delay as potential confounding factors.

### Data source

Participant ʼs records were obtained from KCMC medical birth registry and patient case files from medical records. The KCMC medical birth registry contains data for all women who delivered at KCMC from 2000 to date. These include their social, demographic and obstetric information with their neonates. Patient case files were used to abstract information to estimate time when the decision to conduct emergency caesarean section was done, anesthesia charts were used to determine the time when induction of anesthesia was initiated and time when the delivery of baby occurred. We used times to compute the DDI (as shown in the formula below).

DDI was defined as the time between decisions to conduct a cesarean section to the actual time when the baby was delivered.

The computed DDI that was recorded in minutes as a continuous variable, then it was categorized into two categories (≤75 & > 75 min, respectively).

### Statistical analysis

Data analysis was performed using statistical package for social science (SPSS) version 22. Descriptive statistics were summarized using frequency and proportions for categorical variables. Median and interquartile range was used to calculate the DDI for each diagnosis. A Chi square test was used to determine the associations between a set of variables and DDI, during bivariate analysis Odds ratio (OR) and 95% confidence interval for maternal and fetal outcome associated with DDI were estimated using Logistic regression models. A *P*-value <0.05 was considered statistically significant.

## Results

### Characteristics of the study participants

A total of 598 women who underwent emergency caesarean section during the 9 months study period were legible for the study. Women with previous scar constituted 27.6% (172) of all the participants. Among these, women 9.7% (58) had two previous scars or above and 114(19%) had only one previous scar. Emergency c/sections were conducted for women with 2 or more previous scars who were scheduled for elective c/sections but started labouring while in the ward.

The mean age was 29.25 years (SD 5.63). There was no difference in age, residence, marital status and occupation distribution between those operated within 75 min or those who were operated beyond 75 min (Table 1).

**Table 1:** Characteristics of the study participants (*N* = 598)

Characteristics	DDI	
	≤75 min	> 75 min
Age (years)^a^	29.25(5.63)	
Age group (years)		
< 20	13 (3.59)	7 (2.97)
20–24	66 (18.23)	44 (18.64)
25–29	111 (30.66)	79 (33.47)
30–34	98 (27.07)	58 (24.58)
35–39	61 (16.85)	42 (17.80)
≥ 40	13 (3.59)	6 (2.54)
Residence		
Rural	130 (35.91)	77 (32.63)
Urban	232 (64.09)	159 (67.37)
Marital Status*		
Married	314 (86.74)	197 (83.83)
Not Married	48 (13.26)	38 (16.17)
Level of Education*		
No formal education	7 (1.94)	1 (0.43)
Primary	118 (32.69)	88 (37.45)
Secondary	155 (42.94)	86 (36.6)
College/ University	81 (22.44)	60 (25.53)
Occupation*		
Unemployed	55 (15.19)	36 (15.45)
Employed	307 (84.81)	197 (84.55)
Gestational Age (weeks)		
< 32	13 (3.59)	3 (1.27)
32–36	48 (13.26)	24 (10.17)
37–40	237 (65.47)	172 (72.88)
> 41	64 (17.68)	37 (15.68)

^a^Mean (Standard Deviation), *Missing value

### DDI by indication of emergency caesarean section

Previous scar was the leading cause for emergency caesarean section. Others were poor progress of labor, fetal distress, and hypertensive disorders of pregnancy. The median DDI was 60 min (IQR 40–120 min). CPD has the lowest DDI and patients with imminent threat had DDI between 45 and 60 min (Table 2).

**Table 2:** DDI by indication of emergency caesarean section (*N* = 598)

Indication	n	DDI (minutes) Median (IQ)
Imminent Threat		
Fetal Distress	100	56.5 (37–83.5)
NRFS	38	67.0 (45–172)
APH	29	68.0 (45–139)
Cord Prolapse	2	55.0 (40–70)
Impending Uterine Rupture	2	45.5 (44–47)
Non Imminent Threat		
≥ 2Previous Scar in labour	171	66.0 (43–127)
Poor progress of labor	102	50.0 (38–102)
Hypertensive disorder	88	69.0 (33–159)
PROM	42	61.5 (40–126)
Undiagnosed Breech in labour	22	114.0 (40–172)
Failed Induction	22	59.0 (48–152)
BOH	17	56.0 (30–90)
Big baby	17	70.0 (39–219)
Cervical Dystocia	10	98.0 (45–200)
Prolong Labor	8	214.0 (120–245)
Failure of trial of scar	7	122.0 (61–145)
CPD	3	40.0 (37–100)
Face presentation	2	72.0 (54–90)

### Factors influencing DDI

Hypertensive disorders in pregnancy and prolonged labor had statistically significant influence on prolonged DDI of more than 75 min(*P*-value 0.037 vs. 0.032, respectively).The decision to anesthetic time of more than 30 min and anesthesia to delivery of the baby having more than 10 min significantly influenced prolonged DDI (Table 3).

**Table 3:** Factors influencing DDI (*N* = 598)

Factors	DDI		*P*-value
	≤75 min	> 75 min	
Gestational Age (weeks)			
< 32	13(3.59)	3(1.27)	
32–36	48(13.26)	24(10.17)	0.504
37–40	237(65.47)	172(72.88)	
> 40	64(17.68)	37(15.68)	
Fetal Distress			
No	276(76.24)	184(77.97)	
Yes	86(23.76)	52(22.03)	0.408
APH			
No	344(95.03)	223(94.49)	
Yes	18(4.97)	13(5.51)	0.745
Previous Scar			
No	262(72.38)	164(69.49)	
Yes	100(27.62)	72(30.51)	0.201
Hypertension Disorders			
No	315(87.02)	195(82.63)	
Yes	47(12.98)	41(17.37)	0.037
Prolong labor			
No	360(99.45)	230(97.46)	
Yes	2(0.55)	6(2.54)	0.032
Decision to anesthesia time			
≤ 30	208(57.46)	3(1.27)	
> 30	154(42.54)	233(98.73)	0.000
Anesthesia to delivery of baby			
≤ 10	129(35.64)	71(30.08)	
> 10	233(64.36)	165(69.92)	0.035

### Association between DDI and fetal outcomes

The fetal outcomes that were observed in this study were Apgar score in first and fifth minute, birth weight, transfer to neonate care unit, early neonatal deaths and still births. Since the facility is not capacitated with destructive delivery equipments, cases with stillbirths were operated for obstetric obstructive indication. These outcomes were analyzed in different cut offs of DDI (Tables 4 and 5). A total of 165 babies (28%) were transferred to neonatal unit department. Only 10.3% babies were delivered within 30 min. Half (50%) of the babies who were born between 30 and 75 min were transferred to neonatal unit (Table 4). Neonates born after 75 min, had higher odds of getting Apgar score of less than 7 in the first and fifth minute when compared to other cut off times.

**Table 4:** Association between DDI and Fetal outcome (*N* = 598)

Variables	Transfer to neonatal unit		COR(95% CI)	AOR(95% CI)
	No	Yes		
DDI				
< 30	56(76.7)	17 (23.3)	1	1
30–75	207(71.6)	82(28.4)	1.30(0.72–2.37)	1.29(0.60–2.79)
> 75	170(72.0)	66(28.0)	1.28(0.69–2.36)	0.96(0.39–2.37)
30 min				
≤ 30	56(76.7)	17 (23.3)	1	1
> 30	377(71.8)	148(28.2)	1.29(0.73–2.30)	1.29 (0.60–2.78)
75 min				
≤ 75	262 (72.6)	99(27.4)	1	1
> 75	170(72.0)	66(28.0)	1.03(0.72–1.49)	0.74(0.46–1.20)

AOR: Adjusted for Fetal Distress, APH, Hypertension Disorders, PROM, Decision to anesthesia time and Anesthesia to delivery of baby and weight of child

**Table 5:** Association between DDI and Fetal outcome (*N* = 598)

DDI, minutes	Apgar Score (1st minutes)				Apgar Score (5th minutes)			
	<7	≥7	COR(95% CI)	AOR(95% CI)	<7	≥7	COR(95% CI)	AOR(95% CI)
DDI								
< 30	3(4.1)	70(95.9)	1	1	1(4.8)	72(12.5)	1	1
30–75	20(6.9)	269(93.1)	1.73[0.5–6.0]	2.47[0.50–6.00]	11(52.4)	278(48.2)	2.85[0.36–22.4]	6.19[0.43–88.9]
> 75	19(72.0)	217(28.0)	2.04[0.59–7.11]	2.04[0.59–7.11]	9(42.8)	227(39.3)	2.85[0.35–22.9]	7.72[0.43–138.7]
30 min								
≤ 30	3(4.1)	70(95.9)	1	1	1(4.8)	72(12.5)	1	1
> 30	39(7.4)	486(92.6)	1.29[0.69–2.43]	1.56[0.68–3.59]	20(95.2)	505(87.5)	1.16[0.48–2.79]	1.25[0.41–3.78]
75 min								
≤ 75	23(6.4)	339(93.6)	1	1	12(57.1)	350(60.7)	1	1
> 75	19(8.1)	217(91.9)	1.87[0.56–6.22]	2.29[0.61–8.58]	9(42.9)	227(39.3)	2.85[0.37–21.6]	3.44[0.39–29.9]

AOR: Adjusted for Fetal Distress, APH, Hypertension Disorders, PROM, Decision to anesthesia time and Anesthesia to delivery of baby

(OR: 2.29; 95% CI: 0.61–8.85, vs. OR: 3.44; 95% CI 0.39–29.9) (Table 5) but this were not statistically significant. There were 8 still births, of whom 7 were fresh and one was macerated. There was one early neonatal death due to low score and prematurity. Five stillbirths were operated after 75 min and this was not statistically significant (*P* = 0.45).

### Association between DDI and maternal outcomes

Women operated more than 75 min had 6 times higher odds of losing blood more than 1000mls (OR: 5.79; 95% CI: 0.05–7.18) and 2% higher odds of staying in hospital for more than 4 days compared to those who were operated within 75 min but this was not statistically significant (OR: 1.02; 95% CI: 0.63–1.64), (Table 6).

**Table 6:** Association between DDI and Maternal outcome (*N* = 598)

DDI, min.	Blood Loss (mls)				Hospital Stay (days)			
	<1000	>1000	COR(95% CI)	AOR(95% CI)	≤4	>4	COR(95% CI)	AOR(95% CI)
DDI								
< 30	72(98.6)	1(1.4)	1	1	59(12.6)	14(10.9)	1	
30–75	288(99.7)	1(0.3)	0.25[0.02–4.05]	0.55[0.03–10.3]	231(49.3)	58(44.9)	1.06[0.55–2.03]	0.76[0.36–1.61]
> 75	235(99.6)	1(0.4)	0.31[0.0–4.96]	3.21[0.02–653.9]	179(38.1)	57(44.2)	1.34[0.7–2.58]	0.78[0.32–1.81]
30 min								
≤ 30	72(98.6)	1(1.4)	1	1	59(12.6)	14(10.9)	1	1
> 30	523(99.6)	2(0.4)	0.28[0.02–3.07]	0.55[0.03–10.3]	410(87.4)	115(89.1)	1.18[0.64–2.19]	0.76[0.36–1.62]
75 min								
≤ 75	360(99.4)	2(0.6)	1	1	290(61.8)	72(55.8)	1	1
> 75	235(99.6)	1(0.4)	0.77[0.07–8.49]	5.79[0.05–7.18]	179(38.2)	57(44.2)	1.28[0.86–1.9]	1.02[0.63–1.64]

AOR: Adjusted for Hypertension Disorders, Fetal Distress, APH,Decision to anesthesia time and Anesthesia to delivery of baby

## Discussion

This study found that only 12.3% of the women were operated within 30 min with a median DDI of 60 min. We also found that less time was used to conduct emergency caesarean section compared to other studies which took more 120 min [5–7].Women with imminent threat had shorter DDI. This was consistent with previous study in Ghana [7]. This could be explained by the fact that immediate action was taken to such cases without any form of delay because these patients were within the ward.

The prolonged decision to anesthetic time influenced DDI but factors that caused the delay were not determined as it was a retrospective study. Previous studies conducted elsewhere have reported factors that influenced delay in decision to anesthetic time [4–6, 8, 9].

In the present study, the anesthesia to delivery of the baby was also prolonged. This may be explained by the fact that many patients had previous scars and adhesions can cause delay before extracting the baby. KCMC is also a teaching hospital whereby anaesthetic students get trained for induction of anesthesia which could also prolong the anesthesia to delivery of the baby time.

In the current study, babies with weight of more than 4 kg and risk of infection from premature rupture of membranes (PROM), were more likely to be sent to neonatal unit for observation as per requirements of the hospital protocol. Neonates with difficulty in breathing, low Apgar score and birth asphyxia and still births had DDI more than 75 min. Similarly observations have been reported in England, India, and Nigeria [3, 4, 6, 10].

Our findings also suggest a significant influence on prolonged DDI for hypertensive disease and prolonged labour cases. For hypertensive cases, initial attempts to stabilize blood pressure prior to delivery may contribute to this delay. For prolonged labour cases without imminent threat clinicians may resort to attend indications with imminent threats first due to presence of a single operational theatre.

In this study only blood loss and hospital stay were observed and that may be because most of the patients were clinically stable in the ward and those even who had longer stay of more than 4 days were on antibiotics or anti-hypertensive drugs. However, some previous studies by Chow [8] and Hussein [5] reviewed many other maternal outcomes which were not present in our study.

This study encountered a number of limitations. Detailed information on the intrapartum care of these patients couldn’t be ascertained due to a retrospective nature of the study. Specific type of anesthesia and the other detailed aspects of anaesthetic care couldn’t be retrieved from our database. Moreover, this study didn’t seek to identify specific factors that contributed to the described delays.

## Conclusions

The delivery decision interval for caesarean section at KCMC is 60 min, which is longer than the recommended standard interval. The DDI was influenced by decision to anesthesia time, anesthesia to delivery of baby, prolonged labor and hypertension disorders. A time frame of 75 min can be acceptable after triage and need for urgency have been evaluated.

## Abbreviations

ACOG: American College of Obstetrics and Gynecology
AOR: Adjusted odds ratio
APH: Antepartum Hemorrhage
BOH: Bad obstetrics history
COR: Crude odds ratio
CPD: Cephalopelvic Disproportion
DDI: Decision Delivery Interval
KCMC: Kilimanjaro Christian Medical College
LW: Labor Ward
NRFS: None Reassuring Fetal Status
OBGYN: Obstetrics and Gynecology
p3: Pediatrics III i.e. intensive care unit
PPH: Postpartum Hemorrhage
